# The transcriptional factor ZEB1 represses Syndecan 1 expression in prostate cancer

**DOI:** 10.1038/s41598-018-29829-1

**Published:** 2018-07-31

**Authors:** Nancy Farfán, Nallatt Ocarez, Enrique A. Castellón, Nilo Mejía, Antonio García de Herreros, Héctor R. Contreras

**Affiliations:** 1 Department of Basic and Clinic Oncology, Faculty of Medicine, University of Chile, Santiago, Chile; 2 Institute of Agricultural Research (INIA-Chile), La Platina Research Centre, Av. Santa Rosa 11, 610, P.O. Box 439-3, Santiago, Chile; 3 Programa de Recerca en Càncer, Institut Hospital del Mar d’Investigacions Mèdiques (IMIM), Barcelona, Spain; 4 Departament de Ciències Experimentals i de la Salut, Universitat Pompeu Fabra, Barcelona, Spain

## Abstract

Syndecan 1 (SDC-1) is a cell surface proteoglycan with a significant role in cell adhesion, maintaining epithelial integrity. SDC1 expression is inversely related to aggressiveness in prostate cancer (PCa). During epithelial to mesenchymal transition (EMT), loss of epithelial markers is mediated by transcriptional repressors such as SNAIL, SLUG, or ZEB1/2 that bind to E-box promoter sequences of specific genes. The effect of these repressors on SDC-1 expression remains unknown. Here, we demonstrated that SNAIL, SLUG and ZEB1 expressions are increased in advanced PCa, contrarily to SDC-1. SNAIL, SLUG and ZEB1 also showed an inversion to SDC-1 in prostate cell lines. ZEB1, but not SNAIL or SLUG, represses SDC-1 as demonstrated by experiments of ectopic expression in epithelial prostate cell lines. Inversely, expression of ZEB1 shRNA in PCa cell line increased SDC-1 expression. The effect of ZEB1 is transcriptional since ectopic expression of this gene represses SDC-1 promoter activity and ZEB1 binds to the SDC-1 promoter as detected by ChIP assays. An epigenetic mark associated to transcription repression H3K27me3 was bound to the same sites that ZEB1. In conclusion, this study identifies ZEB1 as a key repressor of SDC-1 during PCa progression and point to ZEB1 as a potentially diagnostic marker for PCa.

## Introduction

Prostate cancer (PCa) occupies the second place in cancer incidence in men worldwide^1^. In PCa, epithelial cells undergo morphological changes, acquiring mesenchymal characteristics, in a process called epithelial to mesenchymal transition (EMT). EMT occurs naturally in events such as gastrulation, neural crest formation and wound healing^2^. Nevertheless, it has been observed an association between EMT and tumor progression^3,4^.

EMT is characterized by a series of changes that impinge epithelial integrity, with the loss of cell to cell adhesion (associated to E-cadherin down-modulation) and apico-basal polarity, altered cell to extracellular matrix (ECM) adhesion, cytoskeleton rearrangements^5^, and increased migration, invasion and apoptosis resistance^6^. Among the stimuli and signaling pathways triggering EMT are the transforming growth factor (TGF-β), fibroblast growth factor (FGF), and epidermal growth factor (EGF), and pathways such as those involving Wnt, Notch, NF-κB, and HIF1/2. All these elements activate transcription factors such as SNAIL, TWIST and ZEB^3,5^, that repress genes maintaining epithelial integrity (being E-cadherin the most relevant) and induce genes related to the mesenchymal phenotype (matrix metalloproteinases and fibronectin)^5,7–12^. SNAIL and ZEB proteins belong to the family of zinc finger type transcription factors that bind directly to the promoter sequences 5′-CACCTG-3′ or CAGGTG (E-box)^13,14^. The increase of these transcription factors has been related to aggressiveness and poor prognosis in carcinomas, like PCa^15^. For instance, in PCa there is a correlation between increased SNAIL levels and the dedifferentiation of the prostatic gland^15,16^. Furthermore, PCa cell lines with increased SLUG show more invasiveness, migration^17^, and aggressiveness, favoring PCa castration resistance^18^. Moreover, a subpopulation of the PCa cell line PC3, with high ZEB1 levels, has shown increased invasive capabilities^19^. Additionally, high Gleason PCa samples displayed higher ZEB1 protein levels than low Gleason samples^20^.

ZEB transcriptional factor family has two highly conserved members: ZEB1 and ZEB2^21^. These have 8 zinc fingers, 4 in the amino terminal domain (C_2_H_2_ type), 1 in the central domain and 3 in the carboxyl terminal domain (C_3_H_3_ type)^22^. ZEB1 interacts with the carboxyl terminal binding protein (CtBP) recruiting co-repressors like histone deacetylases (HDACs), histone methyltransferases, Polycomb repressive complex 2 (PRC2), and BRG1^12,13,22–24^. Accordingly, ZEB proteins work as potent transcriptional repressors of E-cadherin (CDH1) gene and other epithelial proteins^22,24^. ZEB1 is induced during EMT by the coordinated action of SNAIL and TWIST; it has been suggested that ZEB proteins extend and potentiate the repression of epithelial genes initiated by SNAIL^25,26^.

Syndecans (SDCs) are membrane proteoglycans (PG) with a large extracellular domain containing chains of glycosaminoglycans (GAGs) that bind to core proteins^27^, a transmembrane portion and a highly conserved intracellular domain^28^. Furthermore, SDCs have an important role in the adhesion process, and the extracellular domain binds cytokines, growth factors (FGF, EGF) and ECM molecules, such as Fibronectin and Laminin^28,29^. Depending on the extracellular context SDCs cooperate with integrins and modify the adhesion to the ECM^30^. Accordingly, SDCs participate in the regulation of cellular motility, proliferation and differentiation^30^. SDC-1 is expressed mainly in epithelial cells, its distribution is baso-lateral, and influences polarization, morphology and cell positioning^29^.

Several evidences indicate that SDC-1 might be involved in the EMT process. For instance, SDC-1 silencing in epithelial cells induce a mesenchymal phenotype increasing their invasive capacity and decreasing E-cadherin expression^31^. On the contrary, ectopic expression of SDC-1 in murine mammary epithelial tumor cells induce an epithelial morphology, decreasing cell proliferation^32^. Furthermore, the anti-tumoral effects of SDC-1, thus decreased cell proliferation, have been shown in other cancer types, such as myeloma^33^, and PCa^34^. Moreover, the decrease of SDC-1 has been associated with poor prognosis in head and neck^35^, hepatocellular^36^, pulmonary^37^, cervical uterine^38^, breast^39^ and PCa^40^.

Collectively, these evidences encouraged us to analyze if SDC-1 is regulated by EMT transcriptional factors such as SNAIL, SLUG or ZEB1. In this study, we show that SDC-1 is negatively controlled by ZEB1 that binds to the SDC-1 promoter reducing SDC-1 transcription. We also demonstrate that cell adhesion is impaired by ZEB1 ectopic expression.

## Results

### The epithelial to mesenchymal markers and SDC-1 change their expression during the prostate cancer progression

**Figure 1:**
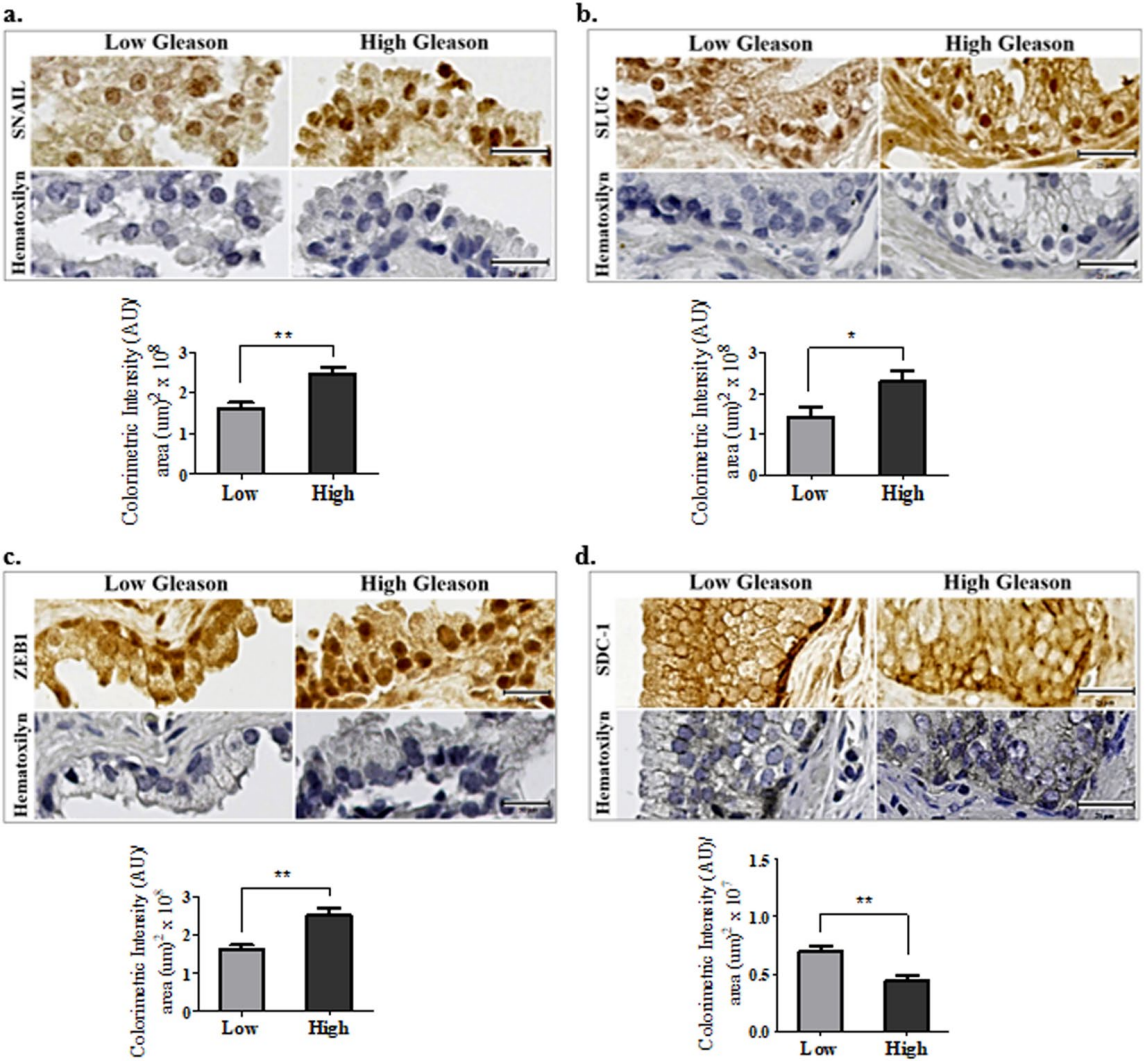
Immunohistochemistry (IHC) in PCa samples with low and high Gleason Score. PCa samples of low Gleason score (2–4) and high Gleason score (8–9) (from 3 patients of low and 3 of high Gleason score) were included in paraffin and serial sections of 5 µm were obtained. 50 photographs were included for the analysis of each immunodetection. *Upper IHC images*, the localization and expression of EMT transcriptional factors: (**a**) SNAIL, (**b**) SLUG and (**c**) ZEB1, were determined in the same samples. Besides was determined the (**d**) SDC-1 expression. One of the serial sections of each sample was utilized to hematoxylin staining as negative control without primary antibody. To SNAIL, SLUG and ZEB1 only were considered the nuclei to quantification. The bars correspond to 25 µm (1000x). *Lower graphs*, the markers levels by colorimetric intensity/area are shown (for details of each marker quantified, see supplementary figures). The data represent the average of three independent experiments (mean ± s.e.m.). T-test statistic analysis was realized, *p < 0.05; **p < 0.01.

All the EMT transcription factors analyzed (SNAIL, SLUG and ZEB1) increased their nuclear expression in PCa epithelial cells (Fig. 1a–c). The SNAIL immunostaining in low Gleason samples was weak, confirming previous data from our laboratory^16^. In high Gleason samples, an increased nuclear staining intensity and a large number of positive cells were observed. SNAIL identified only in the nucleus (Fig. 1a).

SLUG expression was mainly nuclear and stronger than SNAIL in the low Gleason score samples. SLUG intensity in high Gleason score samples was also higher in agreement with results reported by other authors^16,39^. In high Gleason score samples, some cells show a cytoplasmic staining, nevertheless SLUG have no nuclear exportation signal. This finding could be explained to the tissue disorganization in high Gleason score samples (Fig. 1b). ZEB1 location was mainly in the nucleus being higher in high Gleason score than in low score (Fig. 1c). This observation is congruent with data from the literature^20^.

SDC-1 extracellular domain was observed mainly in the basal-lateral region of glandular epithelial cells membrane with higher intensity in the glandular basal zone. SDC-1 staining was higher in low than in high Gleason samples (Fig. 1d). This is in agreement with previous results from our laboratory^16,37,40^.

The quantification of all markers was performed under a specific threshold, to exclude nonspecific labeling (Fig. S1).

**Figure 2:**
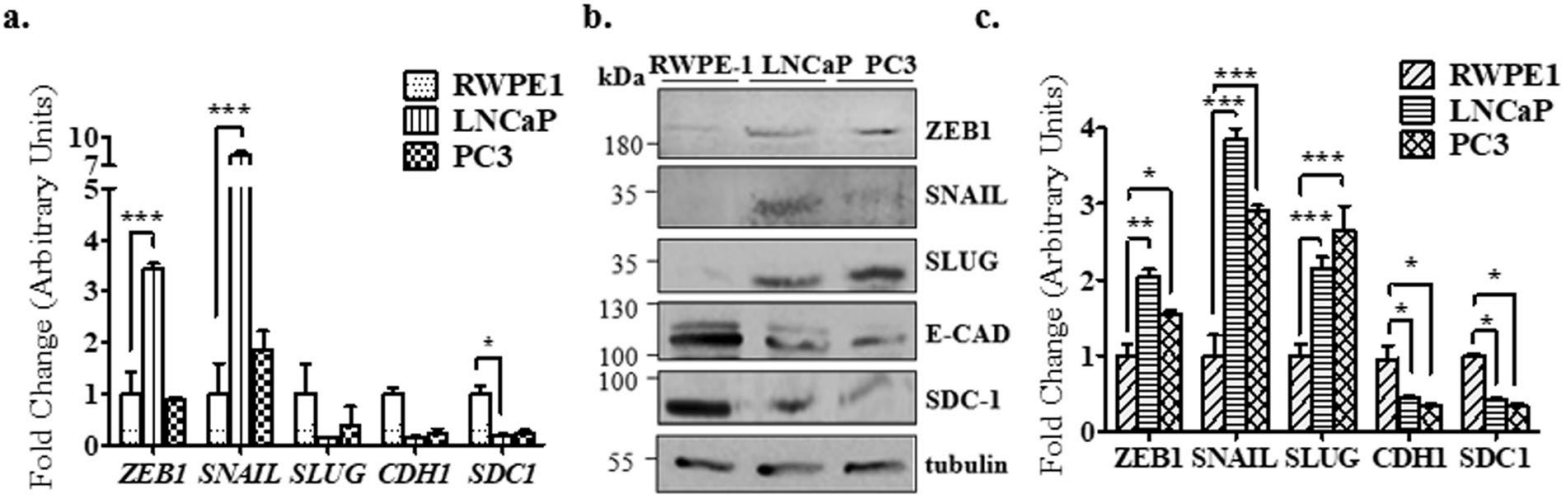
Basal expression of SDC-1 and EMT markers in epithelial and prostate cancer cell lines. (**a**) Real time RT-PCR of basal levels for mesenchymal markers *ZEB1*, *SNAIL, SLUG*, epithelial marker E-cadherin (*CDH1*) and *SDC-1* in the prostate epithelial cell line RWPE-1 and in the PCa cell lines LNCaP and PC3. The expression of each gene was normalized to constitutive gene *PUMILIO*. Afterward, the normalized genes expression levels were normalized to the values obtained from the epithelial cell line RWPE-1.The data represent the average of three independent experiments (mean ± s.e.m.). ANOVA test was used with a Bonferroni post-test, ***p < 0.001 and *p < 0.05. (**b**) Representative Western blot image of EMT markers protein levels: SNAIL, SLUG, E-Cadherina (E-CAD) and SDC-1, using α-tubulin as loading control. (**c**) Relative EMT markers protein levels to α-tubulin by optic density were quantified. The data represent the average of three independent experiments (mean ± s.e.m.). ANOVA test was used with a Bonferroni post-test, ***p < 0.001, **p < 0.01 and *p < 0.05.

Collectively, our data from patients show that in high Gleason score samples there is an increase in EMT transcriptional factors expressions and a decrease in SDC-1 in high Gleason. Therefore, SDC-1 could be regulated by these EMT transcription factors. We determined the basal expression of EMT markers and *SDC-1*, in the epithelial cell line RWPE-1 and in PC3 and LNCaP PCa cell lines. RWPE-1 and PC3 cell lines are cultured in free androgens media, as recommended by ATCC. On the contrary, LNCaP cell line even when culture in presence of androgens is recommended, in this study these cells were cultured in absence of androgens, to maintain high levels of ZEB1 avoiding androgen negative feedback^40–42^ (Fig. 2).

*CDH-1* and *SDC-1* mRNA basal levels were lower in LNCaP and PC3 than in RWPE-1 cells (Fig. 2a). Results from E-cadherin and SDC-1 protein levels were similar to the mRNA levels (Fig. 2b,c). The mesenchymal markers *SNAIL* and *ZEB1* showed high basal levels of mRNA in LNCaP compared to PC3 and RWPE-1 cells (Fig. 2a). However, SNAIL and ZEB1 protein levels were increased in LNCaP and PC3 compared to RWPE-1 cells. Moreover, LNCaP cells showed lower ZEB1 protein than *ZEB1* mRNA levels (Fig. 2a,c). The mesenchymal marker *SLUG* showed low mRNA levels, but high protein levels in LNCaP and PC3 cells, in comparison to RWPE-1 cells, which might be a consequence of other signaling pathways activation (Fig. 2). The differences between mRNA and protein levels of a same marker could be attributed to differences between mRNA transcription and translation processing, mainly in cancer cells^43^.

In summary, these findings demonstrate that mesenchymal markers as SNAIL and ZEB1 are increased in LNCaP and PC3 PCa cell lines, and epithelial markers as E-cadherin and SDC-1 are high only in the RWPE-1 prostate epithelial cell line. These findings suggest a correlation with the expression protein patterns observed by IHC in low and high Gleason samples.

### SNAIL and SLUG ectopic expression induce no change in SDC-1 levels in PCa cells

**Figure 3:**
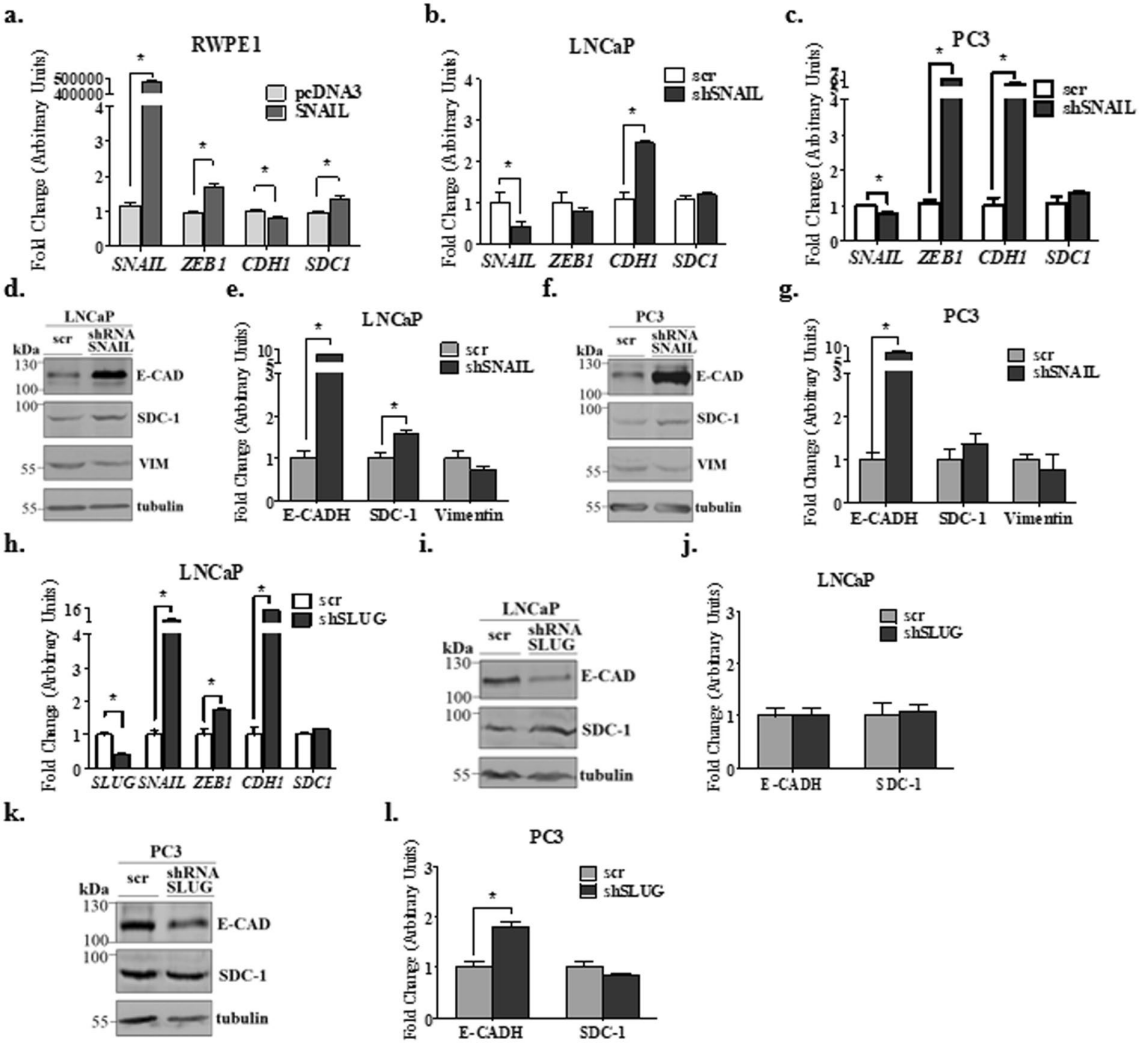
mRNA and protein levels for SDC-1 and EMT markers in prostate cell lines with ectopic expression and silencing of SNAIL and SLUG. (**a**,**b**,**c** and **h**) Real time RT-PCR for the mesenchymal markers: *SNAIL*, *SLUG*, *ZEB1* and the epithelial markers *CDH1* (E-cadherin) and *SDC-1*. The constitutive gene *PUMILIO* was used as normalizer gene in all the experiments. (**a**) The prostate epithelial cells RWPE-1 were transfected with the coding sequence for SNAIL by transient transfection or with the empty vector pcDNA3 as control. Fold change was normalized against the pcDNA3 control. The PCa cell lines: (**b**) LNCaP and (**c**). PC3 cells were transduced by a mix of five shRNA against SNAIL sequence, or the scramble control sequence (scr). Fold change was normalized to the scr control. (**d**–**g**,**i**–**l**) Western blot of EMT markers protein levels: SNAIL, SLUG, E-Cadherin (E-CAD), vimentin (VIM) and SDC-1. The constitutive protein α-tubulin was used as normalizer in all the experiments. Representative western blot images in LNCaP (**d**) or PC3 (**f**) cells transduced with a mix of five shRNA against SNAIL (shRNA) or with a scramble sequence (scr) as control. Relative EMT markers protein levels to α-tubulin by optic density (O.D) were quantified in LNCaP (**e**) or PC3 (**g**) cells with silencing of SNAIL. (**h**) LNCaP cells were transduced with SLUG sequence or with the empty vector (EV) as control. Fold change was normalized to the EV control. Representative western blot image in LNCaP (**i**) or PC3 (**k**) cells were transduced by a mix of five shRNA against SLUG sequence, or the scramble control sequence (scr). Fold change was normalized to the scr control. Relative EMT markers protein levels to α-tubulin by O.D were quantified in LNCaP (**j**) or PC3 (**l**) cells with silencing of SLUG. The data represent the average of three independent experiments (mean ± s.e.m.) in all the graphs. The t-student test was used, *p < 0.05.

We used the epithelial prostatic cell line RWPE-1, that has high *SDC-1* mRNA levels compared to PCa cell lines. SNAIL was ectopically expressed in RWPE1 cells, causing an increase in mesenchymal markers, repression of *CDH-1* and an increase of *SDC-1* mRNA levels (Fig. 3a). This increase of *SDC-1* mRNA levels was unexpected and could be a compensatory mechanism related to epithelial markers repression, such as E-cadherin, occludins or claudins^8,9,44^.

LNCaP and PC3 cell lines were transduced with lentiviral particles for SNAIL silencing (Fig. 3b–g). This showed an increase in *CDH-1* mRNA levels, but *SDC-1* mRNA levels showed no change (Fig. 3b,c). Collectively, these findings indicate that SNAIL has not participation in *SDC-1* mRNA repression. In LNCaP cells, SNAIL silencing resulted in an increase of SDC-1 protein levels (Fig. 3d,e). On the other hand, SNAIL silencing in PC3 cells induced no changes in SDC-1 protein levels (Fig. 3f,g).

LNCaP and PC3 cells were also transduced with lentiviral particles for SLUG silencing (Fig. 3h–l), and this cells showed similar results to those described with the SNAIL silencing. Accordingly, SLUG had no effect in SDC-1 mRNA or protein levels (Fig. 3f–l).

### ZEB1 represses SDC-1 expression in prostate epithelial cells

**Figure 4:**
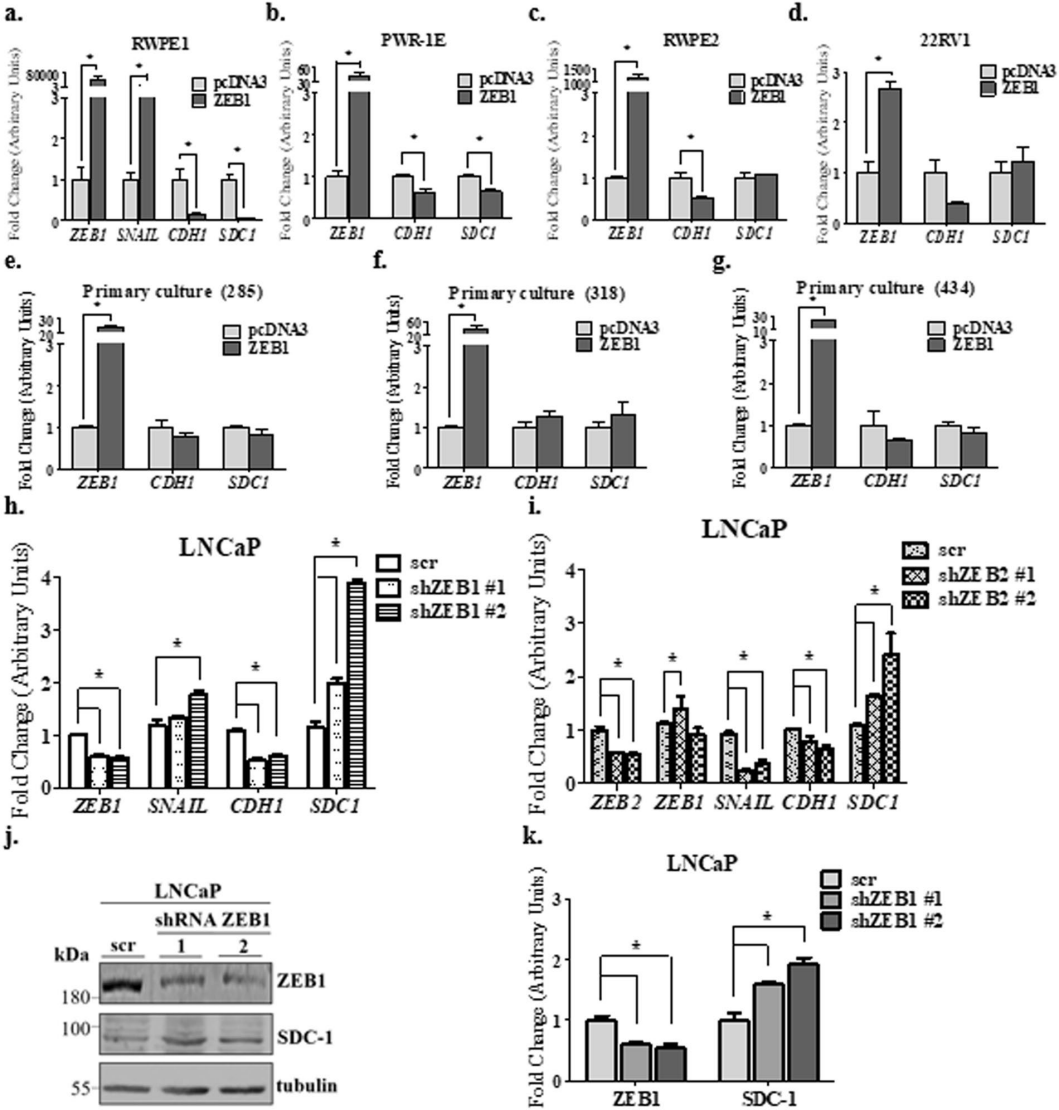
ZEB1 effects on EMT and SDC-1 mRNA and protein levels in prostate cell lines and PCa primary cell culture. Real time RT-PCR for the mesenchymal markers: *SNAIL*, *ZEB1* and the epithelial markers *CDH1* (E-cadherin) and *SDC-1*. The constitutive gene *PUMILIO* was used as normalizer gene in all the experiments. The transient transfection with the coding sequence for ZEB1 or with the empty vector (pcDNA3) (as control) was realized to different epithelial prostate cell lines: RWPE1 (**a**), PWR1E (**b**), RWPE2 (**c**), the prostate primary tumor cell line 22Rv1 (**d**) and epithelial cells from PCa patients called: 285 (**e**), 318 (**f**) and 434 (**g**). Fold change was normalized to the pcDNA3 control. (**j**,**k**) Real time RT-PCR in LNCaP cells transduced with two different shRNAs against ZEB1 (**h**) or ZEB2 (**i**) and a negative scrambled control (scr). Fold change was evaluated with respect to the scr control. (**j**) Representative western blot image in LNCaP transduced with two different shRNAs against ZEB1 and a negative scrambled control (scr). (**k**) Relative EMT markers protein levels to α-tubulin by O.D were quantified in LNCaP cells with silencing of ZEB1. The data represent the average of four independent experiments (mean ± s.e.m.) in all the graphs. The t-student test was used, *p < 0.05(**a**–**g**). ANOVA test was used with a Bonferroni post-test, *p < 0.05 (in **h**,**i** and **k**).

Epithelial prostate cells RWPE1, PWR-1E, RWPE2 (a cell line that lacks p53 expression) and the primary tumor PCa cell line 22Rv1, were transfected with the ZEB1 coding sequence to analyze EMT markers and *SDC-1* mRNA levels (Fig. 4a–d). Only RWPE-1 and PWR1E cells displayed SDC-1 repression in the presence of ZEB1 over-expression (Fig. 4a,b).

The SDC-1 transcriptional repression by ZEB1 was shown in the prostatic epithelial cell lines RWPE-1 and PWR1E, which keep epithelial expression markers, such as the androgen receptor, lack activated oncogenic pathways, are p53+ and were immortalized with a copy of human papilloma virus 18 (HPV-18) and adenovirus 12-SV40 hybrid virus (Ad12-SV40) respectively. In contrast, the prostatic epithelial cell line RWPE-2 (deficient of p53 and tumorigenic), derived from the RWPE-1 and transformed with Ki-ras, using the Kirstin murine sarcoma virus (Ki-MuSV), showed no repression of SDC-1 by ZEB1, suggesting a relationship between RAS signaling and regulation of ZEB1 on SDC-1. Furthermore, in the 22Rv1 cell line, derived from primary tumor and lacking RAS mutations, no SDC-1 repression by ZEB1 was observed. However, these cells carry a mutation in the Ras protein specific guanine nucleotide releasing factor 1 (RASGRF1)^45^. This protein is a guanine nucleotide exchange factor (GEF) that allows GDP/GTP exchange activating the RAS protein. The function of this RASGRF1 mutation has not been determined and it could be related to a high GDP/GTP exchange rate, which might be associated with higher RAS activation. On the other hand, 22Rv1 cells come from PCa primary tumor and are tumorigenic. Thus, RWPE-1 cells and PWR-1E are less aggressive than the RWPE-2 cells, and these in turn, are less aggressive than 22Rv1 cells. These findings suggest that ZEB1 repress *SDC-1* in cells that maintain most of the epithelial prostate characteristics, such as RWPE-1 and PWR1E cells. Even so, the expression of RASGRF1 in 22Rv1 cells was rather weak, 10 times lower than in metastatic PC3 cell line. Therefore, a role of the RAS pathway for the repression of SDC-1 by ZEB1 in 22Rv1 cells remains to be clarified.

In addition, ZEB1 transfection was performed in primary cell cultures from PCa tumor explants. No changes in SDC-1 levels were observed (Fig. 4e–g). Although the ZEB1 over expression in primary cell cultures was lower than in prostate cell lines, because primary cell cultures are less efficient to transfection, this over-expression was sufficient to know if in this cells ZEB1 could be repressed. In addition, this primary cell cultures were from PCa primary tumor, like 22Rv1 cell line (but this last were immortalized) that neither shown a *SDC-1* repression with the ZEB1 over expression (Fig. 4d–g). Subsequently, the ZEB1 silencing in LNCaP cells was approximately 50% in mRNA and protein levels (Fig. 4h,j and k). Nevertheless, the ZEB1 shRNA #1 and #2 increase the *SDC-1* mRNA and protein expression.

The *CDH-1* decreasing in all silenced LNCaP cells could be attributed to other EMT transcriptional factors, because in compensation others repressors with the same target genes could be acting (Fig. 4h,j and k). Also *CDH1* is the main marker repressed by all the EMT transcriptional factors^2,3^.

ZEB1 acts as homo or heterodimer with ZEB2, and both have high homology^19,20^. To identify whether ZEB2 have a similar effect on *SDC-1* mRNA levels, *ZEB2* was silenced in LNCaP cells. Two shRNA against *ZEB2* were efficient in decrease *ZEB2* mRNA levels. In these conditions, *SDC-1* mRNA levels were increased (Fig. 4i). On the other hand, *SNAIL* expression was decreased in all ZEB1 shRNA and *ZEB1* was increased in all of them. Whichever of this EMT transcriptional factors could be involved in the *CDH1* decreasing showed (Fig. 4i). In addition, the over expression of ZEB2 in RWPE1 epithelial cell line displayed a repression of *SDC-1* mRNA levels (Fig. S2) although it was lower than the repression obtained with ZEB1 ectopic expression. In summary, ZEB2 has a similar role to ZEB1 in *SDC-1* mRNA repression.

### SDC-1 promoter activity is repressed by ZEB1

**Figure 5:**
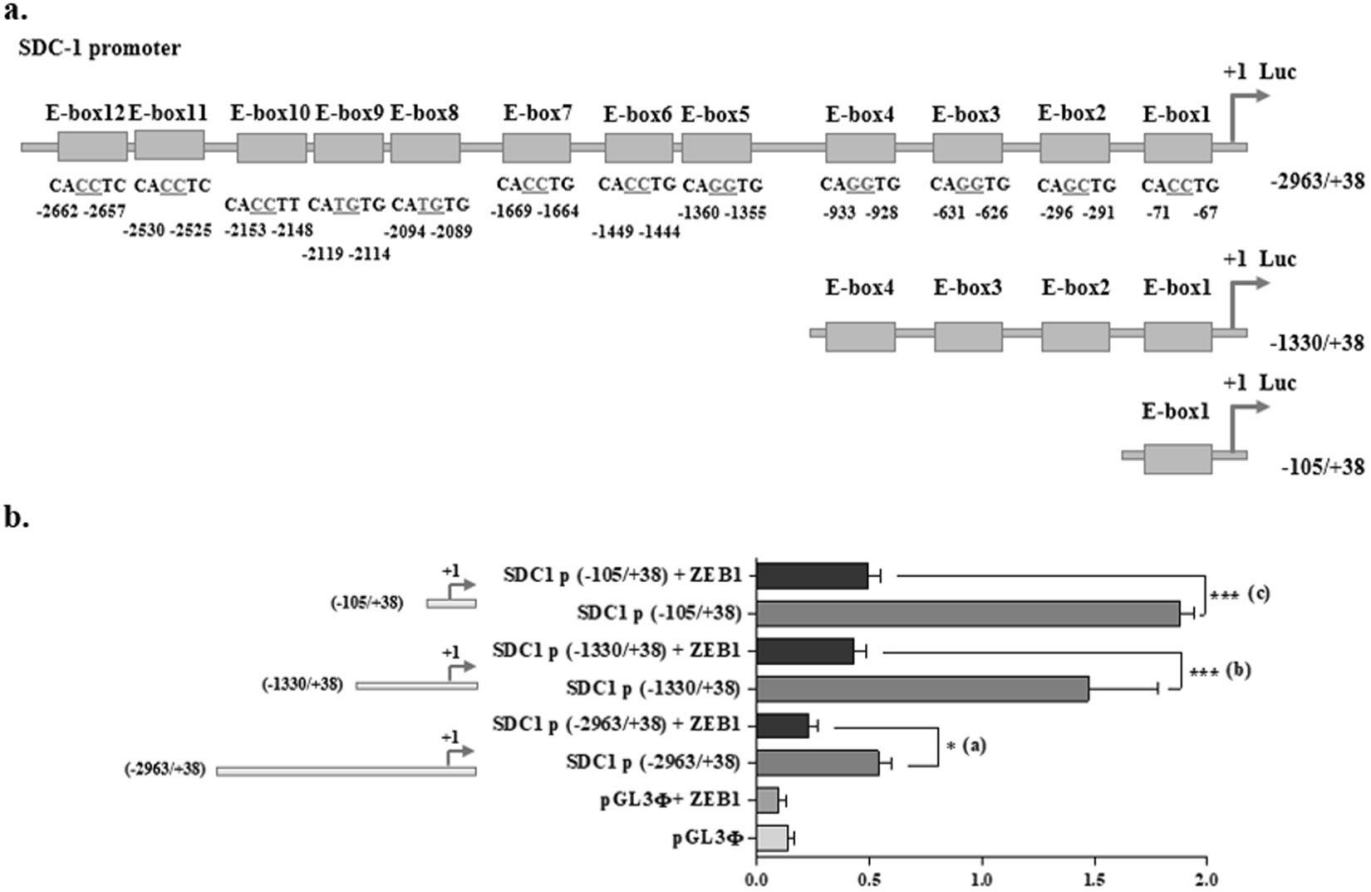
Analysis of SDC-1 promoter activity in RWPE-1 cells with ectopic expression of ZEB1. (**a**) Scheme of the E-Box that could bind ZEB1 or ZEB2 in the human SDC-1 promoter. E-Box sequence correspond to 5′-CANNTG-3′. There are twelve of these sequences in the SDC-1 promoter. The nucleotides composing the different E-box are indicated below them with respect to the transcription starting point (+1). (**b**) Luciferase reporter assay for the SDC-1 promoter containing E-box 1 through 12 (−2963/+38), E-box 1 through 4 (−1330/+38) and E-box 1 (−105/+38). All of these SDC-1 promoter fragments (in plasmid) were transfected in ZEB1 presence (+ZEB1) or absence. As control was quantified the basal luminescence with the pGL3 luciferase empty plasmid (pGL3Φ), in ZEB1 presence (+ZEB1) or absence. The transient transfections were normalized to renilla luciferase by rSV40 plasmid co-transfection. The data represent the average of five independent experiments (mean ± s.e.m.). For each couple of SDC-1 promoter segments (without or with ZEB-1), a t-student test was used, *p < 0.05 (**a**) and ***p < 0.001 (**b** and **c**). Furthermore an ANOVA analysis was realized (*p < 0.05).

The SDC-1 promoter has twelve E-box (Fig. 5a) where EMT promoting transcription factors of the zinc finger family (SNAIL, SLUG and ZEB1/2) could bind^13,14^. SDC-1 promoter activity was analyzed using a luciferase reporter assay in RWPE-1 cells (because these showed high SDC-1 repression in the presence of ZEB1). We used three segments of the SDC-1 promoter, the most extensive includes E-box 1 to 12 (−2968/+38), another segment containing E-box 1 to 4 (−1330/+/+38), and a little segment containing E-box 1 (−105/+38) (Fig. 5a).

To determine the intrinsic activity of these segments of the SDC-1 promoter, the empty pGL3 luciferase plasmid was used as negative control. Each SDC-1 promoter segments displayed an intrinsic activity (Fig. 5b). Less intrinsic activity was observed in the segment with higher number of E-box (E-box 1 to 12 (−2963/+38)), in comparison to the shorter fragments (E-box 1 to 4 (−1330/+38) and E-box 1 (−105/+38)), which was confirmed by an ANOVA analysis (p < 0,05) (Fig. 5b (a)).

After ZEB1 ectopic expression, all three SDC-1 promoter segments showed repression (Fig. 5b). In each SDC-1 promoter segment, the activity of the promoter in presence of the repressor ZEB1was decreased in comparison to the intrinsic activity of the promoter. The t-test analysis was performed for each segment of the promoter and the decreasing between the intrinsic activity and the presence of ZEB1 was higher in the longer fragment of the SDC-1 promoter (E-box 1 to 12 (−2963/+38) (*p < 0.05) (a)), in comparison to the shorter segments of the SDC-1 promoter (E-box 1 to 4 (−1330/+38) (***p < 0.001) (b)) and (E-box 1 (−105/+38) (***p < 0.001) (c)) (Fig. 5b).

These results show that ZEB1 represses SDC-1 promoter activity and that the E-box availability in the plasmid could make them more accessible to ZEB1 than they normally are in the chromatin.

### ZEB1-binding sites of the SDC-1 promoter are coincident with the epigenetic repression mark H3K27me3

**Figure 6:**
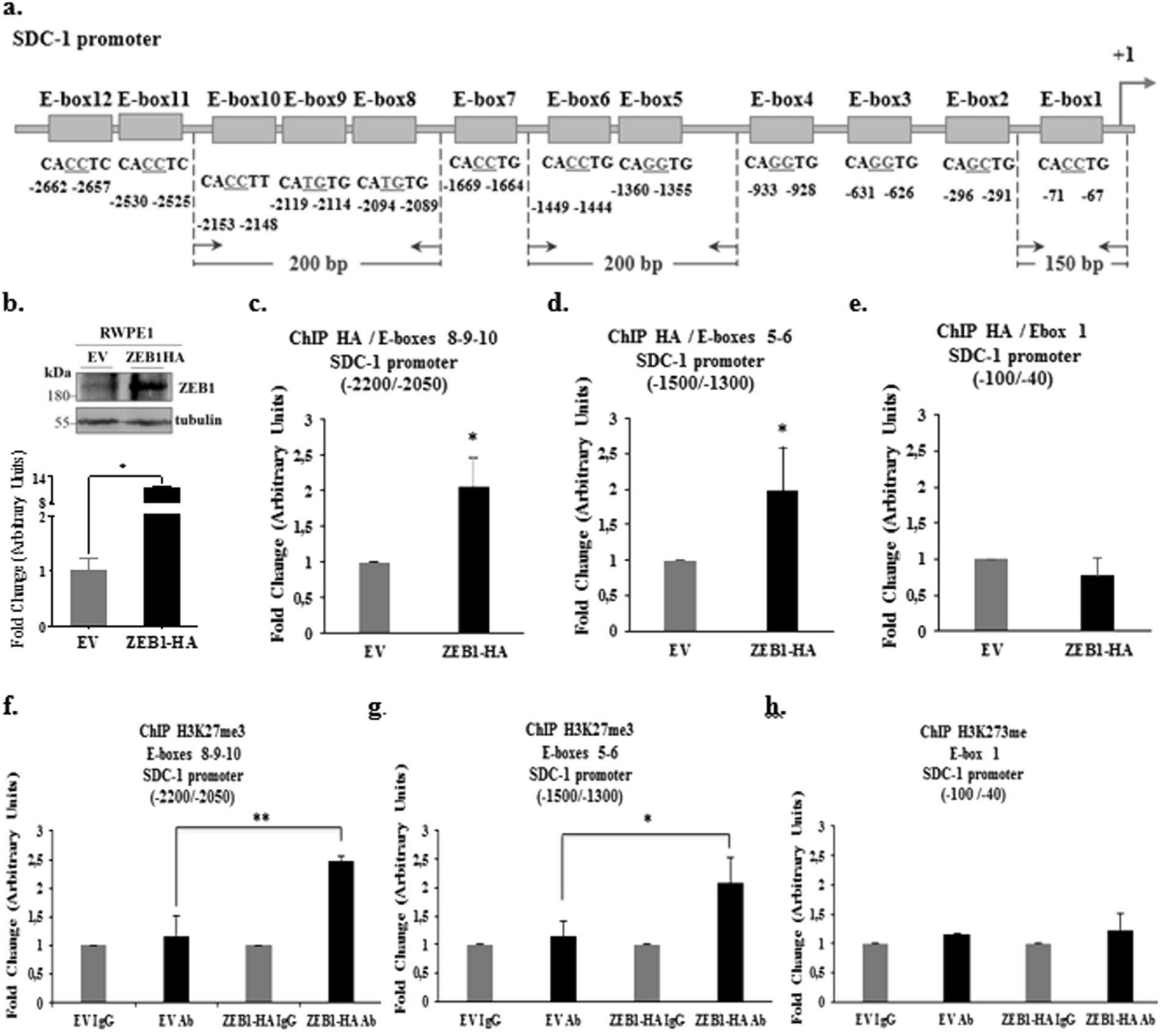
ZEB1 binds to the SDC-1 promoter and is associated with H3K27me3 repression epigenetic mark. (**a**) Scheme of SDC-1 promoter regions analyzed by ChIP. Below the SDC-1 promoter is shown the E-box included in the analysis and the primers are indicated as arrows between the E-box. (**b**) Western blot of ZEB1 in RWPE-1 ZEB1-HA and empty vector (EV) cells. Relative ZEB1 protein levels to α-tubulin were quantified by optic density. The graph show the values normalized to RWPE-1 EV cells. (**c**–**e**) ZEB1 binding to the SDC-1 promoter was evaluated using ChIP in RWPE-1 cells infected with the empty vector (EV) or with ZEB1-HA. Chromatin precipitation was done using the HA epitope and then different regions of the SDC-1 promoter were amplified by real time PCR, E-box 8, 9 and 10 (−2200 a −2050 bp) (**c**), E-box 5 and 6 (−1500 a −1300 bp) (**d**) and E-box 1 (−100 a +40 bp) (**e**). Amplification data obtained by real time PCR was normalized to the data from the chromatin that was not precipitated (input) and expressed as fold change with respect to the EV cells. (**f**–**h**) The repression epigenetic mark H3K27me3 was determined by ChIP in RWPE-1 ZEB1-HA and EV cells. The same SDC-1 promoter regions were analyzed by real time PCR: E-box 8, 9 and 10 (−2200 a −2050 bp) (**f**), E-box 5 and 6 (−1500 a −1300 bp) (**g**) and E-box 1 (−100 a +40 bp) (**h**). Chromatin precipitation was done using a specific antibody to H3K27me3 and as control irrelevant IgG. Amplification data obtained by real time PCR was normalized to the data from the chromatin that wasn’t precipitated (input) and expressed as fold change with respect to the IgG. The graphs showed the average of five independent experiments (mean ± s.e.m.). The t-student test was used, *p < 0.05.

To determine ZEB1 binding to the SDC-1 promoter, the chromatin immunoprecipitation (ChIP) assays was used. Transduction of ZEB1-HA coding sequence was carried out in RWPE1 cells. The over-expression of ZEB1 in transduced ZEB1-HA RWPE1 cells, was verify by western blot, showing that the transduction was effective, increasing two fold the protein levels in ZEB1-HA RWPE1 cells with respect to null cells (Fig. 6b).

Three different sections of the SDC-1 promoter were selected to analyze the possible ZEB1 binding: E-box 8, 9 and 10 (−2500 to −2050 bp), E-box 5 and 6 (−1500 to −1300 bp) and a region with only E-box 1 (−100 to −40 bp) (Fig. 6a). The ChIP assays showed that ZEB1 bound to the SDC-1 promoter in regions with more than one E-box, such as the region with E-box 8, 9 and 10 and the region with E-box 5 and 6 (Fig. 6c,d). The region including the E-box 1 only showed no ZEB1 binding and was used as a negative control (Fig. 6e). In the conformation of the chromatin, ZEB1 binds more readily to two or more E-box near to each other, as described in other promoters^21^.

These findings show that ZEB1 binds to regions with two or more E-box near together. Another important finding was that ZEB1 binds to the SDC-1 promoter in regions distant to the transcription start site.

To determine whether the repressive epigenetic mark H3K27me3 could be associated to the ZEB1 binding in the SDC-1 promoter, a ChIP assay in the RWPE1 ZEB1-HA cells, using an antibody against histone H3 lysine 27 trimethylation (H3K27me3), was performed. The results showed the H3K27me3 in SDC-1 promoter region with E-box 8, 9 and 10 and the region with E-box 5 and 6. The E-box 1 region showed no H3K27me3 mark (Fig. 6f–h), even though the results from the luciferase reporter assay showed that all segments of the SDC-1 promoter were repressed by ZEB1 (Fig. 5b). This result should be taken with precaution, because all the promoter sequences were displayed in a plasmid, without the three-dimensional conformation of chromatin.

### ZEB1 over-expression reduced cell adhesion in epithelial prostate cell lines

**Figure 7:**
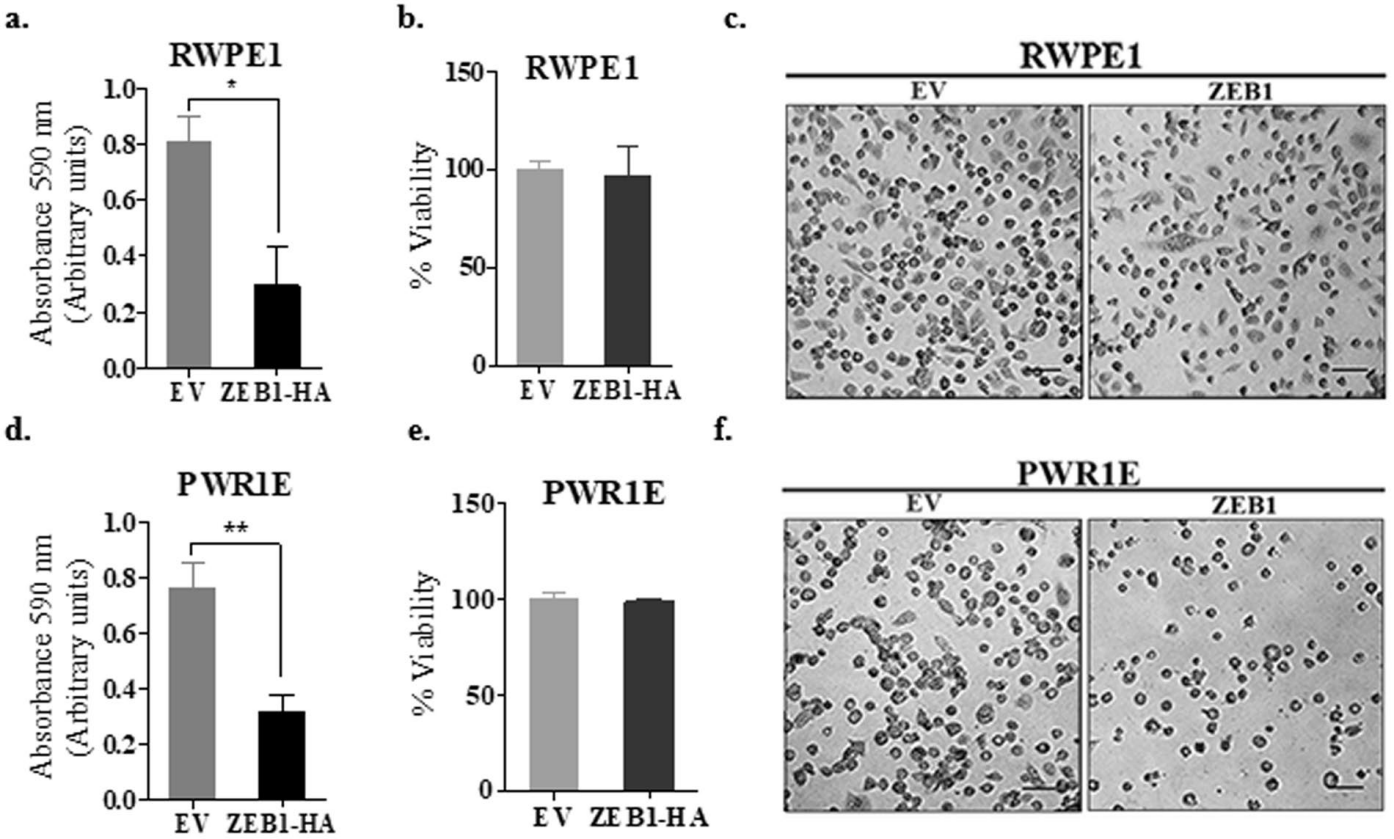
The adhesion of RWPE1 ZEB1-HA and PWRE1 ZEB1-HA cells in extracellular matrix plus collagen I decrease respect to EV cells. Prostate epithelial cell lines RWPE1 (**a**) and PWR1E (**d**) were transduced with ZEB1-HA or empty vector (EV) as control, and then the cells were adhered to extracellular matrix (ECM) plus collagen I for 6 hours. The absorbance was measured to 590 nm and was correlated to the quantity of adhering cells. The cell viability was determined by 3-(4,5-Dimethylthiazol-2-yl)−2,5-Diphenyltetrazolium Bromide (MTT) for RWPE1 (**b**) and PWR1E cells (**e**) transduced with ZEB1-HA or empty vector (EV). The measures of absorbance to 550 nm obtained from EV cells was determined as 100% of viability. Representative image of epithelial cells RWPE-1 (**c**) and PWR1E (**f**) adhered to the ECM plus collagen I. The bar corresponds to 10 µm. The graphs shown the average of three independent experiments (mean ± s.e.m.). The t-student test was used, *p < 0.05.

SDC-1 is a cell-ECM adhesion molecule that binds preferentially to collagen I^36,40^. RWPE-1 and PWR-1E cells with ZEB1 ectopic expression were incubated for 6 hours in an artificial ECM containing collagen I, and then cellular adhesion was determined. Results showed less adhesion in epithelial cells with ZEB1 ectopic expression than in empty vector (EV) cells (Fig. 7a,c,d and f). This effect was not caused by reduced viability as determined by MTT assay (Fig. 7b,e).

## Discussion

This study provides evidence relating the decrease of surface SDC-1 expression and the increase of EMT transcription factors SNAIL, SLUG and ZEB1 in PCa. Previous reports have shown some of these associations for each molecule separately^16,37,39,40,44^. However, in our research all markers were analyzed in serial samples from the same patients, which gives to these correlations a higher value. Nevertheless, there is no report linking the SDC-1 decrease seen in PCa progression to the repressive role of EMT transcription factors. In this work, we demonstrated that ZEB1 represses SDC-1 transcription, by direct binding to its promoter.

It is important to consider in our model, the heterogeneity of PCa cell primary cultures. These cells were originally obtained from the epithelial cells isolated from the PCa samples and might have acquired modifications in the *in vitro* condition, as differential proliferative capacity. Also, primary cultures have low levels of epithelial markers, therefore, changes in some markers such as SDC-1 versus ectopic expression of ZEB1 could be small and difficult to detect. Even though, these PCa primary cultures were used because are better model of epithelial to mesenchymal transition (EMT) than commercial cell lines, supporting the argument that the transcriptional regulation of ZEB1 on SDC-1 is evident in cells with a well-preserved epithelial phenotype. This is important, since depending on the type of cancer and the epithelial marker analyzed, transcriptional repression can continue during tumor progression.

On the other hand, it has been reported that in high Gleason PCa samples there is an inverse correlation between the androgen receptor (AR) and ZEB1 expression, and that there would exist a negative regulation of the AR over ZEB1^30,46,47^. The AR contributes to maintaining the epithelial phenotype, and the loss of the AR in advanced PCa could stabilize ZEB1 expression, and thus maintain the mesenchymal phenotype. According to The Cancer Genome Atlas (TCGA), the expression of SNAIL, SLUG and ZEB1 in PCa patients is high in samples with high Gleason Score compared to those with low Gleason Score, while SDC-1 expression is low in high Gleason samples compared to low Gleason samples^48^. Therefore, our results are concordant with those from TCGA.

In this work we demonstrated that *SDC-1* transcriptional repression is promoted by ZEB1 (and ZEB2). Even more, this ZEB1effect was not observed with SNAIL and SLUG, indicating a mechanism of regulation different to the classical model, where SNAIL and SLUG start the EMT program and later, ZEB1 maintains the mesenchymal phenotype^3,4,25^. In addition, ZEB1 could be exerting a repressive effect over *SDC-1* mRNA levels when the cells still maintain several of prostate epithelial phenotype. This observation suggests that the *SDC-1* mRNA levels decrease could be an independent event at the initial stages of PCa progression.

We propose that SDC-1 repression by ZEB1 occurs mainly in epithelial cells without oncogene or tumor suppressor mutations. Cells acquiring any of these mutations may undergo changes in the signaling pathways triggering the loss of adhesion molecules and other epithelial characteristics or EMT. Therefore, at the time of being immortalized for culture, they may have already presented the lack of these adhesion molecules. In addition, several oncogenic signaling pathways triggering ZEB1 activation have been described. For example, RAS oncogenic pathway induces ZEB1 expression by ERK^25^. On the other hand, the tumor suppressor retinoblastoma (Rb1) represses ZEB1^49^. In primary cultures of mouse embryonic fibroblast (MEFs) the loss of Rb1 promote RAS mutation, activating an axis for tumor initiation^50^. Both, Rb1 and RAS regulate the expression of ZEB1, which in turn induces invasion and metastasis. In the mouse lung cancer model, RAS induces the expression of ZEB1 inducing cancer-initiating cells which are necessary for EMT and metastasis^51^. On the other hand, the p53 tumor suppressor induces miRNA 200, which inhibits ZEB1. With p53 mutations, miRNA 200 decreases conducting to the corresponding ZEB1 increases^52,53^.

With the previous data, we suggest that, before the adhesion molecules loss, mutations in oncogenes or tumor suppressors could have triggered ZEB1 expression of at low levels, favoring transformation of epithelial cells into tumor initiators without necessarily going through EMT, as it may occur in RWPE2 cells. However, the increase in ZEB1 expression of at high level, may have a role in the EMT, which may be the case of 22Rv1 cells. Regarding to the PCa primary cultures shown in this work, they were different from the samples used in the IHC and were thawed from stored stocks. These primary cultures could also have undergone an EMT process. Therefore, they could also have increased the expression of ZEB1 and its function as a transcriptional repressor.

The binding to 2 or 3 E-box, close to each other, in the SDC-1 promoter could indicate that ZEB1 recognizes sequences in this manner, since ZEB1 can homo or hetero-dimerize to bind target sequences^22^. Additionally, ZEB1 and ZEB2 have high homology, differing only in the recruited co-repressors, expression patterns and repressor domain organization, hence, the effect of each one will depend on the cell type^22^.

The SDC-1 promoter sequences bound by ZEB1 are situated far from the transcription starting site. This may be associated with chromatin conformation changes that bring effectors proteins (co-activators or co-repressors) closer to the transcription starting site^54^. Additionally, ZEB1 is less strict in the recognition of the 5′-CANNTG-3′ E-box sequence, however when the nucleotides “NN” are “CC” or “GG”, the transcription factors bind more strongly^55^.

The epigenetic repression mark H3K27me3 was found in the same ZEB1-binding sequences of the SDC-1 promoter (E-box 8 to 10 and E-box 5 and 6). This epigenetic mark is produced by the Polycomb repressive complex 2 (PRC2), which is recruited by co-repressors that bind to transcription factors. ZEB1 has many recruitment sites for co-repressors and co-activators. Among these co-repressors, BRG1 and CtBP have different ZEB1 binding sites. For example, *CDH1* expression can be repressed through both co-repressors, BRG1 or CtBP, that bind to different ZEB1 domains^12,22^. These co-repressors exert their action through the recruitment of chromatin remodelers, such as HDACs and PRC2, which binds to CtBP and carries out the repressive epigenetic mark H3K27me3^22^. Therefore, if CtBP acts as a co-repressor of ZEB1, this repression epigenetic mark could be found at the same ZEB1 binding sites in the SDC-1 promoter.

The role of SDC-1 as adhesion molecule in the cell surface preferentially to collagen I^36,40^, can be suppressed with the shedding of its extracellular domain, a mechanism well described in cancer cells, and mediated mainly by the matrix metalloproteinases (MMPs) 7 and 9^36,43,56^. On the other hand, when RWPE-1 and PWR-1E cells with ectopic expression of ZEB1 were cultured on Collagen 1 the cells decreased adhesion capacity.

In this report, we described a new SDC-1 decrease mechanism, mediated by ZEB1 transcriptional repression in prostate epithelial cells. This decrease in adhesion could be attributed to some of the changes promoted by the EMT induced by ZEB1 ectopic expression, such as cytoskeleton changes, loss of cell polarity and E-cadherin repression, among others. The use of collagen I points to the involvement of a specific type of adhesion molecules like SDC-1. According to the results obtained, this mechanism of decrease in adhesion associated to decrease in surface SDC-1, may occur in the early stages of cellular malignancy, before a significant increase in the membrane receptor shedding. Although it was shown that the ectopic expression of ZEB1 decreases the adhesion capacity in extracellular matrix with collagen I in the prostatic epithelial cells RWPE1 and PWR1E, the functional rescue with SDC-1 was not explored. Indeed, the inclusion of collagen I in the extracellular matrix was aimed for adhesion molecules that bind it with high affinity, as occurs with SDC-1. However, undoubtedly the rescue with SDC-1 is a functional test that would reinforce the repression of SDC-1 by ZEB1. SDC-1 has a function in the adhesion to the extracellular matrix, and its participation in migration would be associated to its interaction with other cell surface molecules, such as integrins, since cell migration is slower in lung epithelial cells that express SDC-1 than in cells silenced for SDC-1^57^.

In summary, our findings strongly support that ZEB1 represses SDC-1 transcription in epithelial prostate cell lines. ZEB1 repression occurs through a direct protein - DNA interaction in the SDC-1 promoter, in areas further away from the transcription start site. SDC-1 transcriptional repression by ZEB1 could occur at an early stage of PCa, when glandular epithelial cells possess high levels of epithelial markers and begin to express mesenchymal markers. In summary, this work contributes to the understanding of SDC-1 regulatory mechanisms during PCa progression and could be seen as a new target for early therapies.

## Materials and Methods

### Tumor specimens

All tumor samples referred in this report derived from patients diagnoses with prostate cancer (PCa) and were obtained after radical prostatectomy at the Clinical Hospital of the University of Chile (CHUCh), after informed consent. The Bioethics Committees of the Faculty of Medicine and CHUCh gave explicit approval to our protocol for tissue collection. In addition, all protocols and handling of hazardous materials were approved by the Risk and Biosafety Unit of the Faculty of Medicine of the University of Chile. Samples from 3 patients of low Gleason score (2 to 4) and 3 patients of high Gleason Score (8 and 9) were used, were obtained serial sections, 50 photos were included for each immunodetection and the posterior quantification. PCa patients had PSA levels from 7, 3 to 38 ng/mL.

### Immunohistochemistry (IHC)

The serial sections of tissues embedded in paraffin (5 μm thick) was stained with hematoxylin or specific antibodies against ZEB1 (ABN285, Millipore, Billerica, MA), SNAIL (#3879, Cell Signaling Technology, Inc., Danvers, MA), SDC-1 (sc5632) and SLUG (sc15391) both from Santa Cruz Biotechnology, Santa Cruz, CA, according to standard procedures. Samples of low and high Gleason score were completely cover and incubated with DAB-Substrate by 5 minutes for all the markers, at room temperature. The expression was evaluated by a pathologist who interpreted the staining as positive or negative. The digital images were obtained using the digital slide scanner NanoZoomer XR (Hamamatsu Photonics, Japan), with a 40X zoom. The capture was carried out in bright field over entire sample at a high resolution (0, 23 µm/pixel). The image exposure and contrast enhancement were uniformly applied. To nuclear evaluation of SNAIL, SLUG and ZEB1, the nuclei were selected and the rest of the image was omitted (more information in Supplementary Fig. 1). The thresholding level for each marker was determined and was uniform to all photos quantified. The quantification was performed using the Image J program and the background was excluded (Fig. S1).

For immunohistochemistry (IHC) of SDC-1, ZEB1, SNAIL and SLUG, three samples of PCa patients were used to obtain serial sections of these samples and evaluate all the markers in each of these patients. In this way the levels of all markers were compared within the same patients. Although a low number of high and low Gleason samples were used, a total of 50 photographs were obtained for each marker of all the samples (equally distributed among the samples of PCa patients). Then the statistics was performed based on the quantification of each marker in the total pictures obtained.

It is important to explain that samples obtained shortly before the immunodetection were used for the IHCs. In addition, these samples were different from those for primary PCa cell cultures, which were thawed from stocks maintained in liquid nitrogen in our laboratory^58^. However, for ectopic expression of ZEB1, SDC-1 mRNA levels were maintained as controls. This might be due to poor transfection efficiency probably because primary PCa cultures may acquire resistance mechanisms for transfection methods.

### Cell culture

All cell lines were obtained from ATCC, and were incubated at 37 °C in a humidified atmosphere with 5% CO_2_. LNCaP (CRL1740) and 22Rv1 (CRL2505) cell lines were cultured in RPMI Medium (GIBCO Life Technologies, Grand Island, NY). PC3 (CRL1435) cell line was cultured in Dubelcco’s modified Eagle’s Medium (DMEM) (#12400-024, GIBCO). Both media were supplemented with 10% FBS, F-12 and penicillin-streptomycin. RWPE-1 (CRL11609), RWPE-2 (CRL11610) and PWR-1E (CRL11611) cell lines were maintained in KSFM Medium, supplemented with Bovine pituitary extract (BPE) (0,05 mg/mL) and epithelial growth factor (EGF) (5 ng/mL)(#17005-042, GIBCO).

### Primary cell culture

Primary cell cultures were established as mentioned previously^58^. Epithelial cells were maintained in DMEM F-12 supplemented with: 7% FBS, 2 µg/mL insulin, 2 µg/mL human transferrin, 10 ng/mL EGF, 200 ng/mL vitamin A and E, 2 ng/mL sodium selenium, 10^−8^ M dihydrotestosterone and 10^−8^ M hydrocortisone) and were maintained at 37 °C and 5% CO_2_.

### Transient transfection

24 h before the transfection the cells were seeded in 6 well plates: RWPE-1, RWPE-2 and PWR-1E (3,5 × 10^5^ cells/well), LNCaP (1,5 × 10^5^ cells/well), PC3, 22Rv1 and primary cultures (3 × 10^5^ cells/well). 5 µg of pcDNA 3.1-SNAIL-pIRES-GFP, pcDNA 3.1-SLUG and pcDNA 3.1 ZEB1 plasmids were used with their respective empty vectors. Lipofectamine (#12566014, Thermo Fisher Scientific, Waltham, MA) with PLUS reagent (#11514-015, Thermo Fisher Scientific) was used for the transfection of RWPE-1, PWR-1E and RWPE-2 cells. Lipofectamine 2000 (#11668019, Thermo Fisher Scientific) was used for the transfection of LNCaP, PC3, 22RV1 and primary cultures. The cells were incubated with the mix of plasmids plus Lipofectamine for 5 h, in antibiotic and supplements free-medium. After, cells were washed and covered with supplemented medium. mRNA levels were analyzed 24 h post-transfection.

### Lentiviral transduction

7, 5 × 10^4^ cells/well were seeded 16 h before transduction. The pLenti suCMV(target sequence)-Rsv(RFP-Puro) were used to SNAIL (NM_005985.3), SLUG (NM_003068.4) or ZEB1 (NM_001128128 with HA epitope) ectopic expression and as control the plasmid without the target sequence. The pLenti-H1-shRNA (SNAIL/or SLUG) #1/#2/ #3-Rsv(RFP-Puro)) were used to silencing and the control with a scramble sequence (scr). These lentiviral particles were purchased from Gen Target Inc., San Diego, CA. ZEB1 silencing was performed using lentiviral particles against five different segments (#70819, #70820, #70821, #70822, #70818, Sigma-Aldrich, St. Louis, MO) and a scr as control. All cells were incubated with lentiviral particles and 5 μg/mL polibrene by 16 h at 37 °C and 5% of CO_2_. After this, cells were washed and selected for 24 h with puromycin at 1 µg/mL for PC3, 2 µg/mL for LNCaP and 0,5 µg/mL for RWPE-1 and PWR-1E.

### RNA extraction and qRT-PCR

RNA was extracted from cells using TRIzol (15596-026, Ambion, Rockford, IL) and cDNA generated using a Reverse Transcription Kit (#600559, Agilent Technologies), according to the manufacturer’s protocol. Quantitative analysis was performed using SYBR Green Real-Time PCR Master Mix Kit (#4472908, Invitrogen, Carlsbad, CA). The experiments were performed in triplicate for each sample and normalized to the housekeeping gene expression *PUMILIO*. The real time thermocycler Stratagene, model Mx3000P was used, using the program MxPro v2.0. The analysis was performed using the ΔΔCt method^59^. PCR was performed using the following primer sets: *ZEB1*, (forward) 5′-TTCACAGTGGAGAGAAGCCA-3′, (reverse) 5′-GCCTGGTGATGCTGAAAGAG-3′; *ZEB2*, (forward) 5′-ATAAGGGAGGGTGGAGTGGA-3′, (reverse) 5′-CGCGTTCCTCCAGTTTTCTT-3′; *SNAIL* (forward) 5′-TTCCAGCAGCCCTACGACCAG-3′, (reverse) 5′-GCCTTTCCCACTGTCCTCATC-3′; *SLUG*, (forward) 5′-CATGCCTGTCATACCACAACC-3′, (reverse) 5′-CTTGGATGAGGTGTCGGATG-3′; *CDH1*, (forward) 5′-GAACGCATTGCCACATACAC-3′, (reverse) 5′-ATTCGGGCTTGTTGTCATTC-3′; *SDC-1*, (forward) 5′-GCCGCAAATTGTGGCTACT-3′, (reverse) 5′-GGTTCTGGAGACGTGGGAATAG-3′; and *PUMILIO*, (forward) 5′-CGGTCGTCCTGAGGATAAAA-3′, (reverse) 5′-CGTACGTGAGGCGTGAGTAA-3′.

### Protein extraction and Western blot

Whole cell protein extracts were obtained using RIPA buffer (Tris-HCl 20 mM, NaCl 150 mM, EGTA 1 mM, NP40 1% v/v, sodium deoxicholate 1% p/v, Na_3_PO_4_ 2,5 mM, β-glycerophosphate 1 mM y Na_3_VO_4_ 1 mM at pH 7,4) with protease inhibitors cocktail. 50 μg of proteins were loaded and separated by SDS-PAGE and transferred to nitrocellulose membrane. Blots were blocked with 5% milk in 0.1% Tween-TBS. Western Blot was performed with the same antibodies used in IHC protocol, and E-Cadherin (#610181, BD Transduction Laboratories, Lexington, KY), Vimentin (ab8978, Abcam, Cambridge, MA), ZEB1 (sc25388) and α-tubulin (sc8035) from Santa Cruz Bitechnology. Bound primary antibodies were detected with HRP-conjugated secondary antibodies (Bio-Rad Laboratories, Hercules, CA) and the EZ-ECL system. The specific mark of the antibodies used has been showed in the Supplementary Fig. S3.

### SDC-1 promoter segments cloning in pGL3 luciferase plasmid

SDC-1 promoter segments were amplified from PC3 genomic DNA (100 ng) using high fidelity DNA polymerase (K2102, KAPA Biosystems). The primers used for amplification were: E-box 8-9-10 (−2200/−2050), (forward) 5′-TTCCGCCCAGGAGAAAACAGAAAAG-3′, (reverse) 5′-CCTTTCCCTGCCTCTCTTACAGC-3′; E-box 5-6 (−1500/−1300), (forward) 5′-GAGAGGTCGAGGCGATTCTCCC-3′, (reverse) 5′-TTTAAAAGTCACTCACGGCCAAG-3′; E-box 1 (−100/+40), (forward) 5′-AACTTGTTCCTCTGCTGTGGATGGC-3′, (reverse) 5′-CACTCCCAACAGCAGTTATGAGCA-3′. This primers have sequences of restriction enzymes KpnI and HindIII (R0142S y R0104S, New England Bioabs). Each SDC-1 promoter segment was sub-cloned in pCR8/TOPO/TA vector (K2520-20, Invitrogen). Plasmidial DNA was extracted through Zyppy™ Plasmid Miniprep Kit (D4036, Zymo Research) and sequenced by Macrogen Inc. (Korea).

### Luciferase reporter assay

Cells were seeded to 2 × 10^5^ cells/well 16 h before of the transient transfection with 150 ng of SDC-1 promoter segment into pGL3 luciferase, or the control pGL3 luciferase empty vector; plus 100 ng of pcDNA 3.1 ZEB1 or control pcDNA 3.1 (empty vector). pRSV-40 plasmid was co-transfected as control. 48 h after transient transfection, the luciferase and renilla luminescence lectures were obtained using Dual Glo luciferase Kit (E2940, Promega), in a luminometer BioTek model SynergyHT.

### Chromatin immunoprecipitation assay

1 × 10^7^ cells were cross-linked with1% formaldehyde. The reaction was stopped using 0,125 M Glycine. After, the cells were lysed, in presence of protease inhibitors cocktail at 4 °C and the chromatin sonicated using Bioruptor (Diagenode) to obtain fragments of 200 bp. The immunoprecipitation was performed with the following antibodies: 5 μg of anti-HA (H6908, Sigma-Aldrich) (500 μg of protein), 5 μg of anti-H3K27me3 (#069-050, Diagenode) (200 µg of protein) and as control an irrelevant IgG (I5006, Sigma-Aldrich). The samples were incubated with Unblocked Protein A beads (C03020002, Diagenode), washed in columns (M1003S, MoBiTec) and incubated for 1 h at 37 °C. 200 mM NaCl solution was used to reverse cross-linking at 65 °C over night. After, the samples were treated with Proteinase K for 1 h at 55 °C, DNA was purified using columns of MiniElute PCR purification Kit (#28006, Quiagen, Valencia, CA) and eluted with DEPC water. The immunoprecipitated SDC-1 promoter fragments were quantified by real time PCR with the following primers: E-box 8-9-10, (forward) 5′-TTCCGCCCAGGAGAAAACAGAAAAG-3′, (reverse) 5′-CCTTTCCCTGCCTCTCTTACAGC-3′; E-box 5-6, (forward) 5′-GAGAGGTCGAGGCGATTCTCCC-3′, (reverse) 5′-TTTAAAAGTCACTCACGGCCAAG-3′; E-box 1, (forward) 5′-AACTTGTTCCTCTGCTGTGGATGGC-3′, (reverse) 5′-CACTCCCAACAGCAGTTATGAGCA-3′.

### Cell adhesion assay

96-well plates were covered with ECM gel (E-1270) plus Collagen type I (10 µg/ml) (C-9879), both from Sigma-Aldrich, and incubated for 2 h at room temperature. The wells were blocked with 1% cell culture-treated serum albumin in 1X PBS for 1 h at room temperature. In each well cells were seeded (7,5 × 10^4^ cells in 100 µL of supplemented medium), previously detached with 0.025% trypsin/2 mM EDTA, or culture medium as negative control. The cells were incubated by 6 h and later fixed with freezer-cold 100% methanol for 10 min. After three washes, 100 μl of crystal violet solution were added (0.5% w/v crystal violet in 20% ethanol) and incubated 10 min. The excess of crystal violet was removed and the plates were gently shaken for 15 min in 200 µL of 100% methanol. 100 µL of the extracted crystal violet were transferred to flat-bottom 96 well plates and the absorbance was measured at 590 nm.

### Viability assay

7.5 × 10^4^ cells/well were seeded in a 96 well plate and incubated for 6 h and 24 h. After the incubation time, 100 μL of 3-(4,5-Dimethylthiazol-2-yl)-2,5-Diphenyltetrazolium Bromide (MTT) solution were added to each well (15 μL of MTT stock solution (5 mg/ml) in 500 μL of Locke solution (24 mM NaCl, 4 mM NaHCO_3_, 5 mM KCl, 10 mM HEPES, 5 mM Glucose, 2,3 mM CaCl_2_ × 2H_2_O, 1 mM MgCl_2_ × 6H_2_O)) and then kept for 2 h at 37 °C in darkness. Afterwards, the solution was removed and 100 μL of DMSO were added to each well. After 10 min the absorbance was measured at 550 nm in a BioTek SynergyHT plate reader.

### Statistical analysis

All experiments were carried out at least three independent times. Graphs representing data express the mean ± s.e.m. Statistical significance was obtained by analysis of variance for repeated measurements (*one-way ANOVA* o *two-way ANOVA*). To compare continuous variables between two groups the *Student’s t test* was used. *p* ≤ 0,05 was considered as statistically significant. All analysis was done using the GraphPad Prism 5 program.

### Data availability

All data generated and analyzed during the current study are available from the corresponding author on reasonable request.

### Guidelines

All methods and procedures used in this study were in accordance with relevant national and international guidelines and regulations.

## Electronic supplementary material

Supplementary Information
