# Supplementary material for: The transcriptional factor ZEB1 represses Syndecan 1 expression in prostate cancer
Source: Sci Rep. 2018 Jul 31;8:11467. doi: 10.1038/s41598-018-29829-1 (PMC6068163; doi:10.1038/s41598-018-29829-1)
Supplement: Supplementary file 1 — Supplementary Information [file 41598_2018_29829_MOESM1_ESM.docx]

**The transcriptional factor ZEB1 represses Syndecan 1 expression in prostate cancer**

Nancy Farfán^1^, Nallatt Ocarez^2^, Enrique A. Castellón^1^, Nilo Mejía^2^, Antonio García de Herreros^3, 4^ & Héctor R. Contreras^1*^

**^1^**Department of Basic and Clinic Oncology, Faculty of Medicine, University of Chile, Chile. **^2^**Institute of Agricultural Research (INIA-Chile), La Platina Research Centre, Av. Santa Rosa 11, 610, P.O. Box 439-3, Santiago, Chile. **^3^**Programa de Recerca en Càncer, Institut Hospital del Mar d'Investigacions Mèdiques (IMIM), Barcelona, Spain. **^4^**Departament de Ciències Experimentals i de la Salut, Universitat Pompeu Fabra, Barcelona, Spain.

***Corresponding author:**

e-mail: hcontrer@med.uchile.cl

**Identification of quantified region in Immunohistochemistry (IHQ) in PCa samples with low and high Gleason score**

**a. b.**


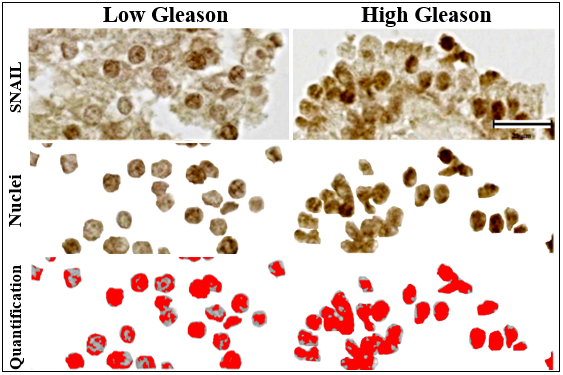

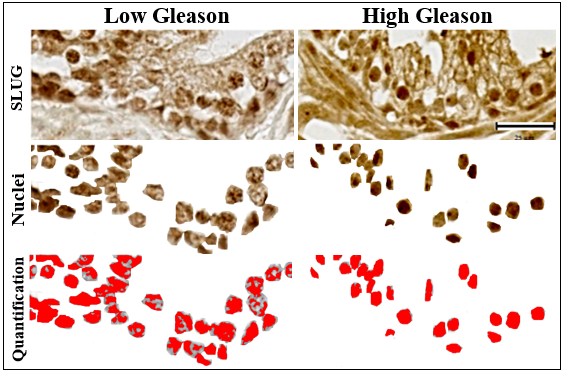


**c. d.**


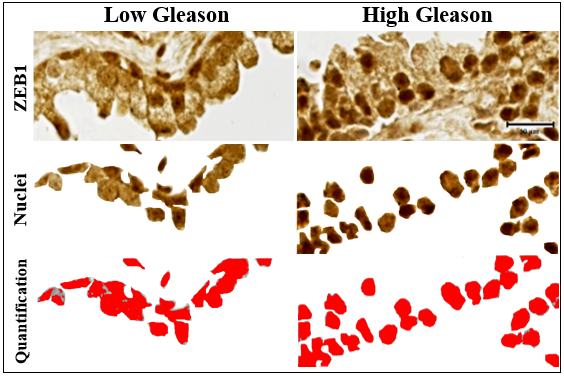

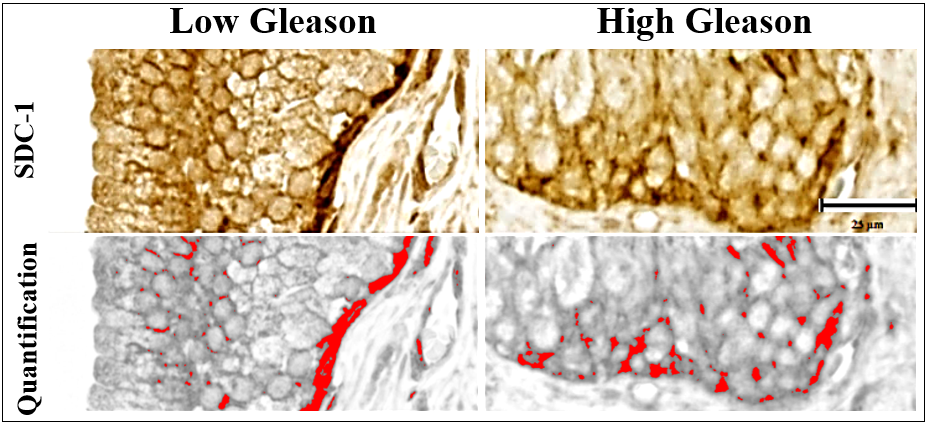


**Supplementary Figure 1. Identification of quantified region in Immunohistochemistry (IHC) in PCa samples with low and high Gleason score.** PCa samples of low Gleason score (2-4) and high Gleason score (8-9) (from 3 patients of low and 3 of high Gleason score) were quantified by Image J. 50 photos were included for the analysis of each immunodetection. In red is shown the specific mark for each antibody: **a.** SNAIL, **b.** SLUG, **c.** ZEB1 and **d.** SDC-1, that was quantified as colorimetric intensity / area. To SNAIL, SLUG and ZEB1 were considered the nucleus to quantification. The red specific mark excluded the background. The bars correspond to 25 µm (1000X).

**ZEB2 effects on EMT markers and *SDC-1* mRNA levels in prostate epithelial cell line RWPE-1**

**Supplementary Figure S2. ZEB2 effects on EMT markers and *SDC-1* mRNA levels in prostate epithelial cell line RWPE-1**. RT-qRT-PCR with ZEB2 transient transfection and empty vector (pCS2) as control. The constitutive gene *PUMILIO* was used as normalizer gene*.* Fold change was evaluated with respect to the empty vector (pCS2) control. The data represent the average of four independent experiments (mean ± s.e.m.). The t-student test was used, *p < 0.05.

**Complete blots relative to the selected images showed in the main Figures**


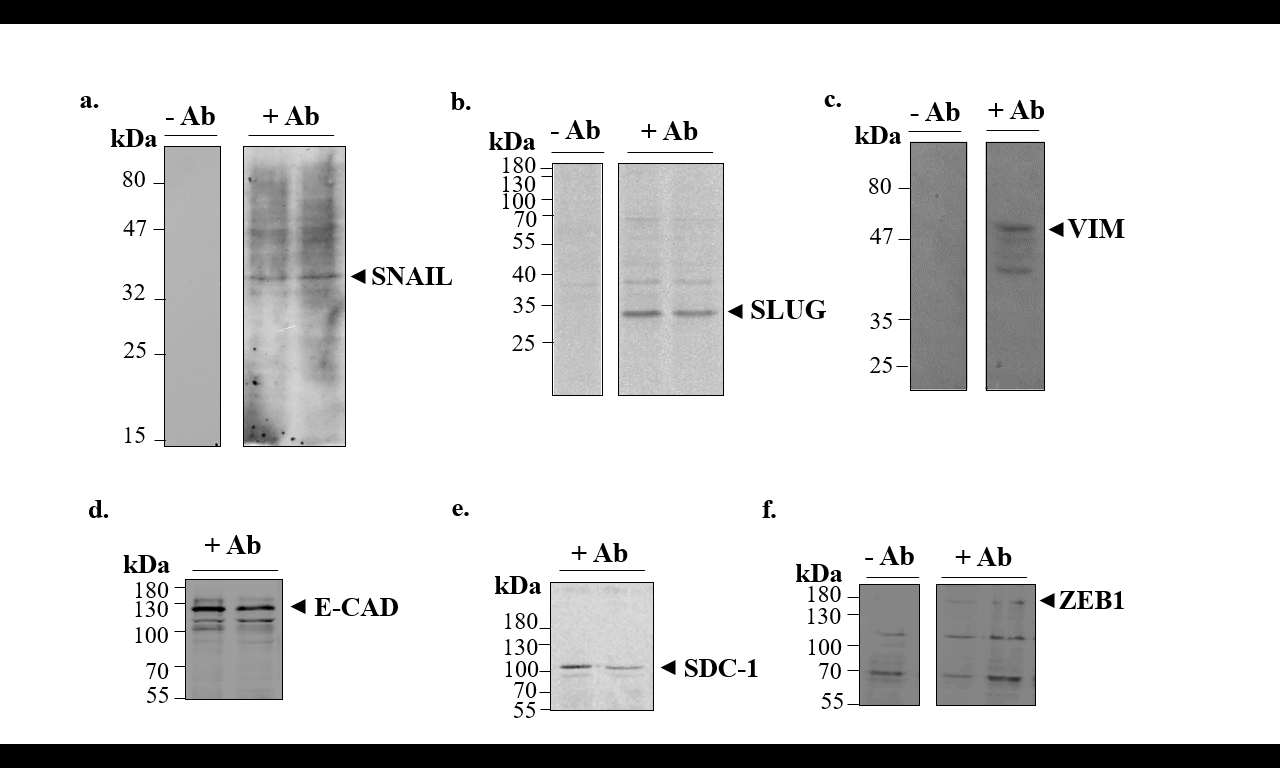


**Supplementary Figure S3**: Western blot showing the specific band for different antibodies: A) anti-SNAIL, B) anti-SLUG, C) anti-vimentin (VIM), D) anti-E-cadherin (E-CAD), E) anti-syndecan-1 (SDC-1), F) anti- ZEB1. Were loaded 50 µL of total protein extracts of PC3 cells. – Ab: control without primary antibody, + Ab: Western blot with primary antibody.

**Consent for publication**

Not applicable.

**Competing interests**

The authors declare that they have no competing interests.
